# Mercury's Northern Rise Core‐Field Magnetic Anomaly

**DOI:** 10.1029/2021GL094695

**Published:** 2021-09-02

**Authors:** Alain M. Plattner, Catherine L. Johnson

**Affiliations:** ^1^ Department of Geological Sciences The University of Alabama Tuscaloosa AL USA; ^2^ Department of Earth, Ocean and Atmospheric Sciences University of British Columbia Vancouver BC Canada; ^3^ Planetary Science Institute Tucson AZ USA

**Keywords:** Mercury, core magnetic field, local magnetic field method, MESSENGER, Northern Rise, Slepian functions

## Abstract

We use magnetic field data collected in orbit around Mercury by the MErcury Surface, Space ENvironment, GEochemistry and Ranging satellite, to detect a regional magnetic field anomaly that is spatially associated with Mercury's Northern Rise topographic signature. Regional spectral analysis indicates a source depth at or below the core‐mantle boundary, and hence the anomaly is of core, not crustal, origin. This observation supports previous studies linking the Northern Rise to a deep‐seated gravity anomaly and reveals connections among core, mantle, and crustal dynamics, likely enabled by Mercury's thin mantle.

## Key Points

- We discovered a magnetic anomaly that coincides with Mercury's Northern Rise topographic feature
- The source depth of this magnetic anomaly is close to Mercury's core‐mantle boundary
- Together with previous research, this indicates links among core, mantle, and crustal dynamics

## 1 Introduction

Mercury is among the few terrestrial planetary bodies in our solar system with an active core magnetic field. Data collected by the MErcury Surface, Space ENvironment, GEochemistry and Ranging (MESSENGER) mission (Solomon et al., 2001) revealed that the internal magnetic field is symmetric with regard to Mercury's rotation axis but asymmetric with respect to the geographic equator (Anderson et al., 2011, 2012; Oliveira et al., 2015; Thébault et al., 2018), sometimes referred to as “an offset axial dipole, OAD” (Anderson et al., 2011, 2012). In addition, evidence for crustal magnetization was discovered in low‐altitude MESSENGER data (Johnson et al., 2015), allowing the determination of crustal field structure north of 35°N at wavelengths less than ∼500 km (Hood, 2015, 2016; Hood et al., 2018; Johnson et al., 2018; Oliveira et al., 2019).

Little is known about Mercury's magnetic field of either core or crustal origin, at spatial scales between the largest scale lengths characterized in the crustal field and the OAD. This is a consequence of external magnetic fields that have wavelengths of ∼1,000 km (Johnson et al., 2012, 2018; Anderson et al., 2014, 2018). Although the spatial patterns of these fields are organized in local time (i.e., in the Mercury solar orbit frame, MSO), their temporally averaged pattern can alias into the geographic frame (i.e., Mercury body‐fixed frame, MBF) because of Mercury's 3:2 spin‐orbit resonance and zero obliquity. Furthermore, mid‐to‐high northern latitude external fields resulting from field‐aligned currents increase in amplitude with decreasing altitude, mimicking the altitude‐dependent behavior of internal fields (Anderson et al., 2014, 2018; Johnson et al., 2018). One study has concluded that nonaxisymmetric contributions to the field comprise only ∼1% of the power in the field at spacecraft altitude, but without a specific focus on the spatial structure of these contributions (Thébault et al., 2018).

Here, we explicitly study non‐zonal, that is, nonaxisymmetric, structure in Mercury's internal field by first estimating and subtracting external fields (Section 2.1). Because the available data only cover mid‐to‐high northern latitudes, we used local methods to calculate an internal magnetic field and its source depth (Section 2.2). We optimized our inversion approach and tested its reliability using synthetic tests (Section 2.3). Finally, we discuss our results (Section 3) in the context of current knowledge regarding Mercury's internal structure (Section 4).

## 2 Methods

**Figure 1:**
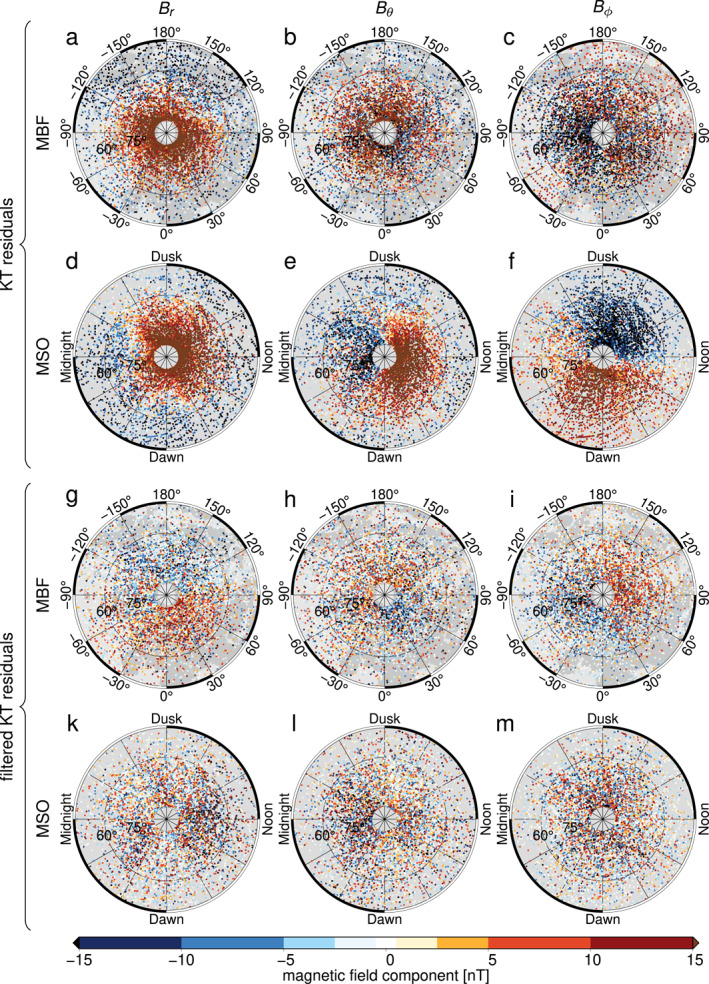
MESSENGER 60 s averaged data (magnetic disturbance index ≤ 30, altitude 200–600 km) after subtraction of the magnetospheric model by Korth et al. (2017), the “KT residuals” in (a–c) Mercury body‐fixed (MBF) and (d–f) Mercury solar orbit (MSO) frame, and (g–m) after empirical external field removal, the “filtered KT residuals” in the (g–i) MBF and (k–m) MSO frame. Left column shows the radial component, center column the colatitudinal component, and right column the longitudinal component. Data in this and in later figures were subsampled by a factor of two for plotting. Maps in this and later figures are stereographic projections from 45°N to the pole.

We used 60 s averaged vector magnetic‐field data from MESSENGER's orbital mission, 2011–2015. We retained orbits with a magnetic disturbance index (Anderson et al., 2013) less than 30, keeping only magnetospherically quiet data. We first subtracted the predictions of a magnetospheric model that accounts for the OAD, magnetopause, and magnetotail fields (Korth et al., 2017) from the data. All three magnetic components of the residual signals, referred to as the KT residuals, showed large‐scale structures organized in the MSO frame (Figures 1d–1f) but not in the MBF frame (Figures 1a–1c). These patterns are consistent with magnetic fields originating from the northern dayside cusp (cf. Figures 1d and 1e with Johnson et al., 2018) and from field‐aligned currents (cf. Figure 1f with Anderson et al., 2014).

### 2.1 External Field Estimation and Removal

Field‐aligned currents constitute magnetic sources in the data region, and so the KT residuals cannot be directly inverted using potential field methods (e.g., Anderson et al., 2011; Blakely, 1995). We grouped the KT residuals in the MSO frame into 100 km altitude bins (measured above Mercury's mean radius of 2,440 km) and fitted and subtracted vector Slepian functions (Plattner & Simons, 2014), constructed for the northern hemisphere of Mercury and band‐limited by maximum spherical‐harmonic degree $L_{\text{ext}}=20$. This corresponds to fields with spatial scales > 900 km at an altitude of 400 km and hence captures the dominant wavelengths of the field‐aligned currents (Anderson et al., 2014). We used those Slepian functions that had more than 50% of their energy within the northern hemisphere. Our results varied only minimally for different choices of $L_{\text{ext}}$ (cf. Figures 1g–1i with Figure S1). For the longitudinal component $B_{\phi }$, we used purely toroidal vector Slepian functions, because these most closely resemble the expected magnetic fields caused by field‐aligned currents. For the radial component, we used radial vector Slepian functions and for the colatitudinal component we used non‐toroidal tangential vector Slepian functions.

The filtered data (KT residual minus empirical external fields) lack regional‐scale magnetic fields in the MSO frame (Figures 1k–1m) but these are visible in the MBF frame (Figures 1g–1i), at altitudes less than 600 km. Data below 200 km contain substantial contributions from short‐wavelength signals of crustal origin. Unlike the signals seen here (Figures 1g–1i), the longest wavelength, largest amplitude signals in the crustal field occur in and around the Caloris region (Hood et al., 2018; Johnson et al., 2018). We therefore retained data between 200 and 600 km altitude. The spatial patterns in all the three components (Figures 1g–1i) are consistent with being the radial, longitudinal, and colatitudinal derivatives of the same potential field.

### 2.2 Inversion Approach

Our goals are to invert for a spherical‐harmonic description of the internal magnetic field from the filtered data (Figures 1g–1i) and to calculate a local power spectrum to estimate a magnetic source depth. Because of Mercury's zero obliquity, axisymmetric fields will be identical in the MSO and MBF frames. Thus, no regional‐scale axisymmetric structure remains after the filtering step in Section 2.1. We inverted for local spherical‐harmonic models using a non‐zonal implementation of the altitude‐cognizant Slepian functions (Plattner & Simons, 2017) for maximum spherical‐harmonic degree $L_{\text{inv}}$ in the region bounded by latitudes 60°N and 84°N. To test our spherical‐harmonic models on an independent data set, we used only the radial and the longitudinal components of the filtered data in our inversions and compared the model‐predicted colatitudinal component with the unused colatitudinal filtered data.

We obtained source depths by minimizing the misfit between a source‐depth‐dependent localized analytical power spectrum, and the local power spectra we obtained from our local models. We constructed the localized analytical power spectrum from the global non‐zonal power spectrum of Langlais et al. (2014) using the localization method of Wieczorek and Simons (2005, 2007), with Slepian functions constructed only from non‐zonal spherical harmonics. We used a tapering bandwidth of $L_{\text{tap}}=3$, providing one well‐concentrated taper within our region and used the eigenvalue weighting of Dahlen and Simons (2008) to calculate the local multitaper spectra.

### 2.3 Synthetic Data Experiments

**Figure 2:**
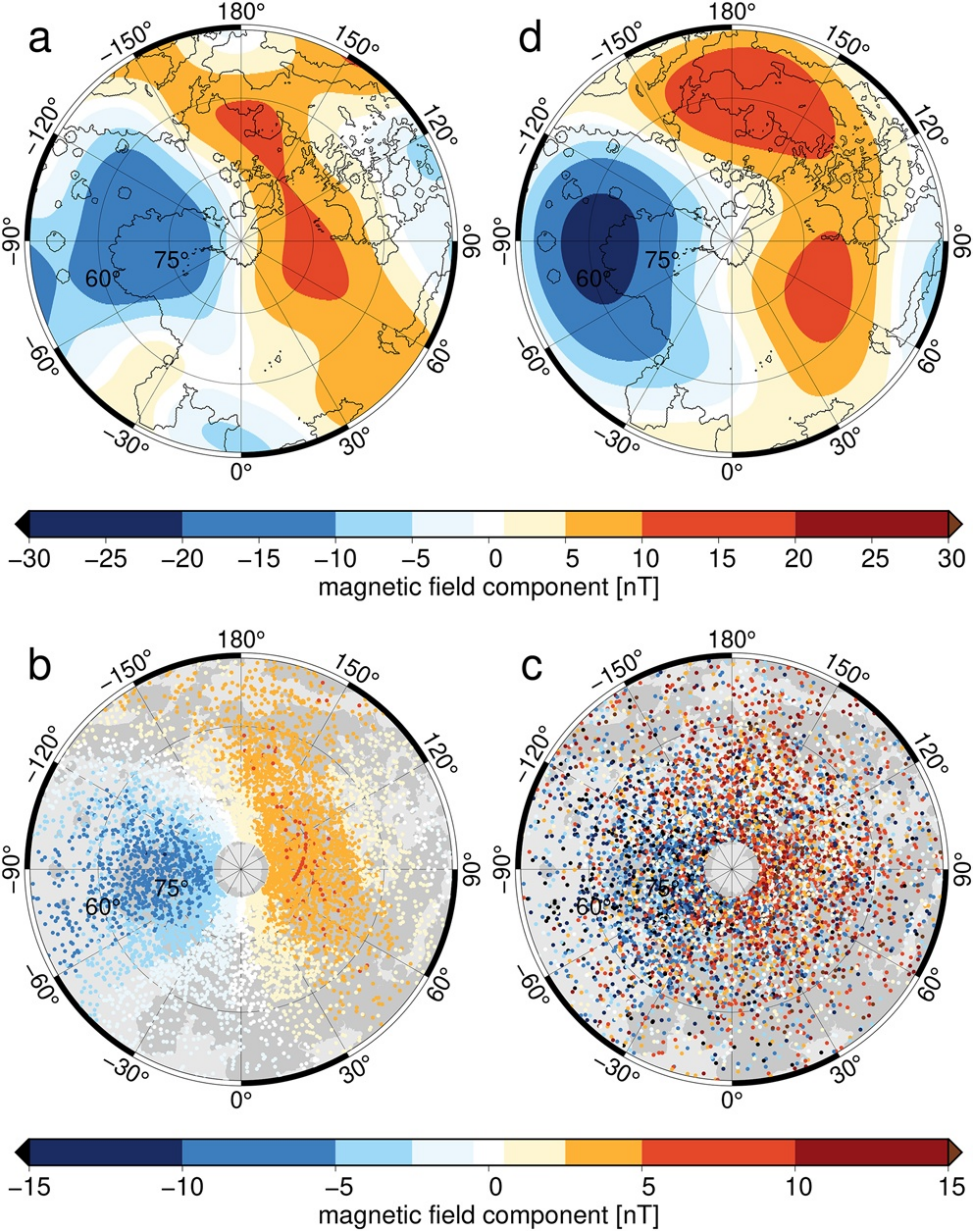
Synthetic experiment setup. (a) Synthetic model with maximum spherical‐harmonic degree 20 created for source radius 2,000 km, radial component plotted on Mercury's surface (r = 2,440 km). (b) Radial component at MESSENGER data locations calculated from (a). (c) Data in (b) with Gaussian random noise added. (d) inversion result from data in (b) using regularization parameter J = 37, radial model component plotted on Mercury's surface (r = 2,440 km). Inversion result is plotted only for spherical‐harmonic degrees ≤ 10 (see text).

Calculating a magnetic‐field model and its corresponding source depth from the spatially limited and noisy data (Figures 1g–1i) posed a formidable challenge. To determine optimal inversion parameters and to demonstrate that recovery of a source depth from these data is indeed possible, we conducted synthetic tests as follows. We specified a non‐zonal magnetic field model following the spectrum by Langlais et al. (2014) for a chosen source depth. We used a source radius of 2,000 km, corresponding to a magnetic source close to Mercury's core‐mantle boundary (Bertone et al., 2021; Genova et al., 2019). The model (Figure 2a) was evaluated at our data locations (Figure 2b) and Gaussian noise was added (Figure 2c) with a standard deviation 2.5‐times that of the evaluated model to obtain synthetic data with characteristics similar to the filtered MESSENGER data (Figure 1g). We tested our source depth estimation procedure (Section 2.2) on these synthetic data for $L_{\text{inv}}=20$ and removed spherical‐harmonic degrees >10 from the resulting model (Figure 2d), for reasons detailed below.

**Figure 3:**
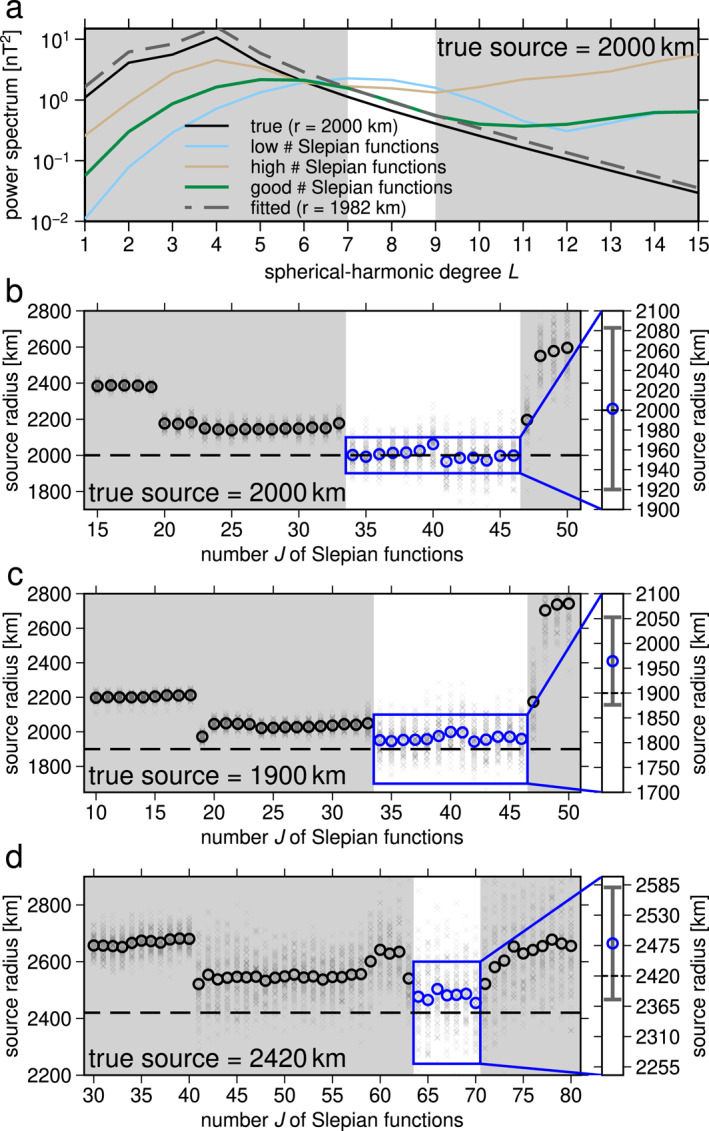
Source depth calculation from synthetic data. (a) Effect of regularization parameter J (number of Slepian functions) on the resulting power spectrum. (b) Source radii for synthetic data with source radius 2,000 km (Figure 2c). Each cross indicates the source radius of one resulting magnetic model with equal‐area random data subselection (50%). Circles represent the mean for each J. The blue box indicates optimal candidates for J (see text). Right panel shows the mean and standard deviation for all results for the optimal range for J and is centered at the chosen source radius. (c) Same as (b) but for synthetic data with chosen source radius 1,900 km. (d) Same as (b) but for chosen source radius 2,420 km (Figure S3), corresponding to a crustal source.

Local spectra obtained from the synthetic data (Figure 2c) using optimal regularization matched the input model power spectrum for spherical‐harmonic degrees 7 to 9 (Figure 3a). The narrow bandwidth results from the limited spatial coverage together with a small signal amplitude relative to the noise. Spherical‐harmonic degrees 7 to 9 correspond to spatial angular diameters, Θ, of 38° to 48° ($\Theta =360{}^{\circ}/\sqrt{L\left(L+1\right)}$). These fit well within the angular diameter 60° of our region and are representative of the spatial scales observed in the filtered data (Figures 1g–1i) as well as in the synthetic data (Figure 2c). Spherical‐harmonic degrees ≤6 correspond to angular diameters of 56° and larger and are thus too large for their spatial power to be reliably resolved. Signals of higher spherical‐harmonic degrees attenuate more with altitude than their lower‐degree counterparts and have therefore a lower signal‐to‐noise ratio at satellite altitude. Here, specifically, signals of spherical‐harmonic degrees ≥10 are substantially affected by noise. Hence, we removed these signals in maps of our models.

We obtained source depths of our magnetic models by spectral misfit minimization (Section 2.2) to spherical‐harmonic degrees from 7 to 9 (Figure 3a). The magnetic models and their local power spectra depend on the choice of inversion regularization parameter J, the number of altitude‐cognizant Slepian functions used (Plattner & Simons, 2017). Strong regularization (too few Slepian functions) prevents the model from fitting the spatial variation in the data and the resulting power spectrum is dominated by the power spectra of the Slepian functions themselves. Too weak regularization (too many Slepian functions) causes the model to fit noise. In both cases, the resulting spectrum is too white, thus the source radius is overestimated compared to an optimal choice for J (Figure 3a). The steepest spectrum and hence the lowest source radius provides the best estimation. We confirmed this with numerical tests as follows.

We calculated source depths for a range of regularization parameters J and for equal‐area random subselection (50%) of the synthetic data (Figure 2c). This was repeated 100 times for each value of J. The source depths obtained varied substantially, but the averages for each J grouped around discrete values (Figure 3b). As expected from our previous discussion, the lowest average source radii were closest to the true source radius for the synthetic model. This guided our choice of an optimal range for J of 34 to 46. Synthetic experiments for source radii set to 1,900 km (Figure 3c) and 2,420 km (Figure 3d) yielded the correct source depth within one standard deviation, distinguishing between core and crustal sources (Figures 3b and 3c versus Figure 3d).

## 3 Results

**Figure 4:**
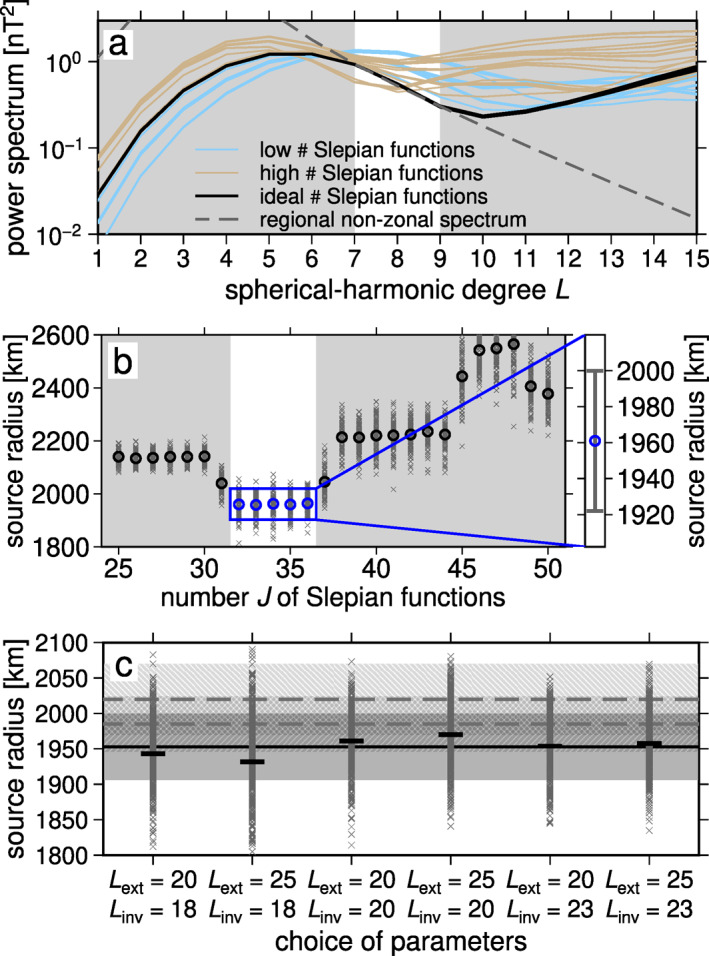
Source depth determination from MESSENGER data. (a) Power spectra obtained for a range of choices of the regularization parameter, J. (b) Each gray cross indicates an inferred source depth for equal‐area random subsampling of 50% of MESSENGER's radial ($B_{r}$) and longitudinal ($B_{\phi }$) data. (c) Resulting source radii using the approach shown in (b) for a range of maximum spherical‐harmonic degrees for data filtering, $L_{\text{ext}}$ and for the maximum spherical‐harmonic degree of the internal field inversion, $L_{\text{inv}}$. Solid line and gray area indicate the mean and one standard deviation for the magnetic anomaly source radius. Dashed lines denote means and hatched areas show standard deviations for the core‐mantle boundary (Bertone et al., 2021; Genova et al., 2019).

We used the same approach as for our synthetic experiments (Section 2.3), to calculate source depths that fit the filtered KT residuals. The power spectra for the filtered MESSENGER data (Figure 4a) behaved similarly to the synthetic data (Figure 3a). Optimal regularization values were J=32 to 36 (Figure 4b). To test the dependence of our results on our parameter choices, we repeated our calculations for a range of $L_{\text{ext}}$ and $L_{\text{inv}}$ (Figure 4c) and obtained a mean source radius of 1,953 km with a standard deviation of 47 km over all $L_{\text{ext}}$ and $L_{\text{inv}}$ and optimal J. The consistency of our source depths confirmed the robustness of our result (Figure 4c) and places the source of the observed field at or below Mercury's core‐mantle boundary (1,985 km ± 39 km Genova et al., 2019; 2,020 km ± 50 km Bertone et al., 2021, and see the discussion by Steinbrügge et al. [2021]).

**Figure 5:**
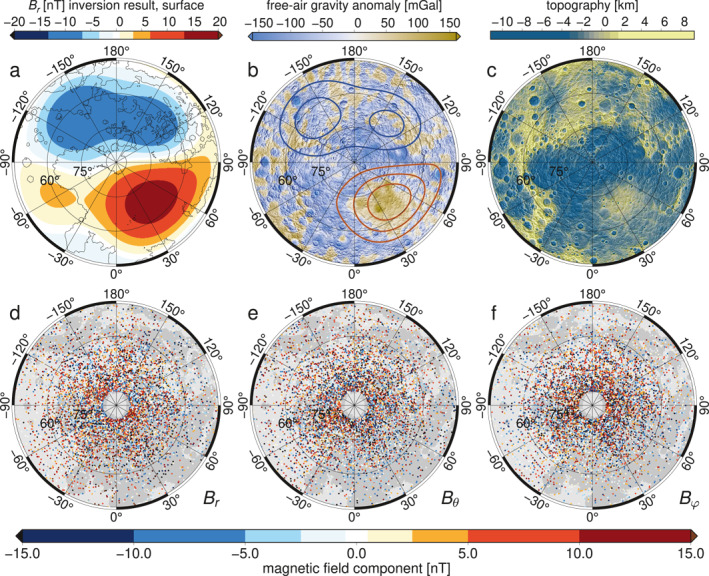
Mercury magnetic field spatial pattern, interpretation, and data residuals. (a) Radial component of the resulting magnetic field, expanded to spherical‐harmonic degree 10, for J = 32 evaluated on Mercury's surface (r = 2,440 km). (b) Mercury's free air gravity anomaly (Genova et al., 2019) with contours (5 nT interval) of the field shown in (a). (c) Mercury's topography (Zuber et al., 2012). (d–f) Residuals after subtracting the evaluated data for the model in (a) from the radial, colatitudinal, and longitudinal data (Figures 1g–1i).

The spatial model radial components for J=32 to 36 (Figure 5a, S2a–S2d) show a positive radial magnetic anomaly between longitudes 30°W and 90°E and latitudes 50°N and 80°N, and a negative anomaly in the complementary longitudes. Model residuals (Figures 5d–5f) calculated as filtered data (Figures 1g–1i) minus the model (Figure 5a) evaluated at the MESSENGER data points contained no obvious regional structure in the MBF frame (Figures 5d–5f). The radial component mean value in the quadrant of the positive radial anomaly was reduced from 4.1 nT in the filtered data (Figure 1g) to 0.5 nT in the model residual (Figure 5d), indicating that the signal was successfully modeled. The complementary longitudes in the filtered radial data and the corresponding regions for the other components showed similar results (Figures 5d–5f).

## 4 Discussion and Conclusions

One other published study has investigated structure in the core field beyond the OAD, using magnetic field data from all MESSENGER orbits and an internal/external field separation approach that assumed a source‐free region (Thébault et al., 2018). In contrast, here we used only magnetospherically quiet data and accounted for the presence of magnetic sources. Our estimated external fields have an RMS signal of 15–17 nT in each field component between latitudes 60°N and 84°N. Because the residual internal fields are relative to the subtracted axisymmetric background fields we cannot, for example, distinguish a positive radial signal between 0°E and 90°E, from simply a less negative background signal. In either case, the data (Figures 1g–1i) and the resulting model (Figure 5a) show that a radial‐field contrast is positive over the Northern Rise relative to its longitudinally averaged background.

Were the observed Northern Rise magnetic anomaly (Figures 1g–1i and 5a) of crustal origin, then we would also expect strong shorter‐wavelength signals to be present. However, although a smooth anomaly ∼450 km in scale of ∼6 nT at 40 km altitude has been inferred to be a crustal field structure correlated with the gravity field (see Figure 5 of Hood et al., 2018), at lower altitudes and in magnetization models, the crustal field signals are much more localized (Figures 5.18 and 5.19 of Johnson et al., 2018). Geological processes typically create magnetic fields with a continuous range of spatial scales, and furthermore, large‐area homogeneous magnetization rarely occurs in nature. These observations, together with our source depth results (Section 3) firmly establish a core versus crustal origin for the field structure inferred here. Thébault et al. (2018) estimated that the non‐zonal part of Mercury's core field at data altitude is ∼1% of the RMS signal, and we find a similar result of 1.1%. At the planetary surface and the core‐mantle boundary (CMB), non‐zonal fields contribute 1.3% and 3% of the RMS signals, respectively. The top of the source region inferred here for the non‐zonal field is up to 170 km below the CMB. It is thus compatible with, but does not require, a stably stratified layer at the top of the outer core, a topic to which we return in this discussion.

We considered whether the southern edge of the Northern Rise magnetic anomaly could indicate the inner edge of the dynamo tangent cylinder (Glatzmaier & Roberts, 1995). The behavior of Earth's field at high latitudes has been proposed to reflect outer core dynamics inside versus outside the tangent cylinder (e.g., Cao et al., 2018; Gubbins & Bloxham, 1987; Olson & Aurnou, 1999) and was suggested to be a possible diagnostic of the tangent cylinder at Mercury (Stanley et al., 2007). In this case, the radius of the inner core, $r_{i}$, would be related to the colatitudinal extent of the non‐zonal signal (Δθ) by $r_{i}=\sin\left(\Delta \theta \right)\cdot$ 2,440 km = 1,220 km, consistent with the results of Genova et al. (2019) and Steinbrügge et al. (2021). The spatial overlap of the magnetic anomaly and the Northern Rise (Figures 5a–5c) would thus be fortuitous. However, the southern boundary of our model is governed by the resolution in the MESSENGER data set. Hence, we do not favor this interpretation.

We explore mechanisms that could explain the spatial association of the core‐field magnetic anomaly with the topographic dome of the Northern Rise. The origin and current support of the Northern Rise topographic feature is still enigmatic. Studies of gravity and topography concluded that the Northern Rise topography is supported by mantle buoyancy, possibly deep‐seated (James et al., 2015; Kay & Dombard, 2019), suggesting a connection of the surface feature to the lowermost mantle properties and dynamics.

First, we consider possible effects of lateral variations in electrical conductivity in the lowermost mantle on the core magnetic field. One possible source of such variations could be variations in the thickness of a postulated FeS layer (Hauck et al., 2013). James et al. (2015) suggested that the relaxation of a locally thinner FeS layer can produce a long‐lived mantle upwelling supporting the Northern Rise topography. The thinner FeS layer would have a reduced vertically integrated conductivity relative to the surrounding regions resulting in less shielding of the core field. Alternatively, variations in the lowermost mantle conductivity, coupled with time variations in the internal dipolar field can produce smaller‐scale induced fields as has been proposed for the Earth during magnetic reversals (Costin & Buffett, 2004). Unlike fields induced by temporal changes in the external field (Johnson et al., 2016; Katsura et al., 2021; Wardinski et al., 2019), these fields would be fixed in the MBF frame. Although secular variation could not be robustly detected by comparing Mariner 10 and MESSENGER data, upper bounds of a few nT/yr are possible (Johnson et al., 2018; Philpott et al., 2014).

Second, we consider lateral variations in thermal properties of the lowermost mantle. Presently, Mercury's mantle is likely not convecting globally (Guerrero et al., 2021; Hauck et al., 2018; Michel et al., 2013; Tosi et al., 2013), however, localized convective cells can form and persist in a mantle with a subcritical Rayleigh number (Solomatov, 2012). Locally enhanced heat flow into the mantle would produce locally enhanced core cooling and could also provide deep support of the Northern Rise. In the absence of a thick stably stratified layer at the top of the outer core, heat flux variations that are asymmetric about the planet's rotation axis can not only produce localized magnetic anomalies, but can also decrease the overall planetary dipole moment (Sreenivasan & Jellinek, 2012). For Mercury, some, but not all (Cao et al., 2014), dynamo models invoke a several‐hundred‐km‐thick stably stratified layer at the top of the outer core to explain the weak strength and relatively large axial quadrupole to dipole ratio of the global field (Christensen, 2006; Christensen & Wicht, 2008; Takahashi et al., 2019; Tian et al., 2015). This is in contrast to the inference here that the top of the source region for the non‐zonal field is less than 170 km below the CMB. An interesting issue then is whether local‐scale fields with source regions close to the CMB could be compatible with a deep‐seated dynamo overlain by a thick globally averaged stably stratified layer. One possibility is that lateral variations in the thermal structure of the CMB could set up regional‐scale flow and/or locally enhanced iron snow production and hence locally enhanced electrical conductivity near the top of a thick stably stratified layer. (Local) double diffusivity may be another mechanism to explain magnetic‐field generation through convection in an otherwise stably stratified layer (Manglik et al., 2010).

The magnetic anomaly associated with the Northern Rise offers a new perspective on the internal workings of Mercury. The apparent link between the magnetic core field, local mantle upwelling, and lithospheric uplift may be a unique feature of Mercury's thin mantle and active core field. Future magnetic field data from the BepiColombo mission will allow the assessment of a regional scale structure in the core field globally.

## Supporting information

Supporting Information S1

## Data Availability

Software developed for the presented research is available at https://www.doi.org/10.5281/zenodo.4768336. This software uses the packages available at https://www.doi.org/10.5281/zenodo.598177. The resulting model of this research is available at https://www.doi.org/10.5281/zenodo.4768373. MESSENGER data were obtained from the Planetary Data System (Korth & Anderson, 2016).
