# Supplementary material for: Mercury's Northern Rise Core‐Field Magnetic Anomaly
Source: Geophys Res Lett. 2021 Sep 2;48(17):e2021GL094695. doi: 10.1029/2021GL094695 (PMC9285016; doi:10.1029/2021GL094695)
Supplement: Supplementary file 1 — Supporting Information S1 [file GRL-48-0-s001.pdf]

Supporting Information for "Mercury's Northern Rise Core-Field Magnetic Anomaly"

A. M. Plattner[1]and C. L. Johnson[2,3].

[1]Department of Geological Sciences, University of Alabama, Tuscaloosa, AL 35487, USA

[2]Department of Earth, Ocean and Atmospheric Sciences, University of British Columbia, Vancouver, BC V6T 1Z4, Canada

[3]Planetary Science Institute, Tucson, AZ 85719, USA

Contents of this file

1. Text S1 to S2
2. Figures S1 to S6

Introduction

This supplementary information contains figures that were not essential to the main article but may provide supplemental confirmation and additional information for interested readers. We demonstrate that the choice of the filtering parameter for the data does not affect the observe spatial pattern (Fig. S1 and Fig. S2b). We also show the spatial pattern of the resulting model evaluated at the core-mantle boundary (Fig. S2b),

---

Corresponding author: A. M. Plattner, Department of Geological Sciences, University of Alabama, Tuscaloosa, AL 35487, USA

and the model for our synthetic crustal-field source depth inversion (Fig. S3). Finally, we show that a completely independent filtering approach yields the same data pattern (Figs. S4–S6).

Text S1.

To demonstrate that our method can calculate the source depth of magnetic fields with spatial patterns substantially different from those shown in the main article Figs 2a,d and 5a, we also show the spatial pattern of the synthetic crustal magnetic field (Fig. S3), for which we successfully found the source depth (main article, Fig. 3d).

Text S2.

In Figs. S4–S6, we show the results of an alternative filtering approach, to demonstrate the robustness of the resulting signal. First we show the KT residuals for a slightly different choice of disturbance index (disturbance index less than 50, (Fig. S4), binned in two altitude bins in the MBF frame. As discussed in the main text, although the residuals appear to have some coherent structure in the MBF frame, they are better organized in the aberrated local time frame. We fit a smooth surface to the KT residuals in that frame (see Fig. S5 caption), fitting each magnetic field component separately in the two distinct altitude bins. This signal is then subtracted from the KT residuals and the data rotated back into the MBF frame. The filtered signals (Fig. S6) show very similar spatial patterns and amplitudes to those obtained from our approach in the main text, most notably a positive radial field anomaly over the Northern Rise. Furthermore, here we see that indeed this filtered signal increases in amplitude with decreasing spacecraft altitude. The filtering

approach in Fig. S5 results in slightly more localized signals than seen in Fig. 1 (main text), although we made no attempt here to adjust the smoothing to match the specific wavelength content of the vector slepian approach in the main text. We chose to adopt the approach described in the main paper rather than the simple median and surface fitting approach in Fig. S5 because the former is based on a mathematical description consistent with the fields originating in a region in which current sources flow.

References

Anderson, B. J., Johnson, C. L., Korth, H., Winslow, R. M., Borovsky, J. E., Purucker, M. E., ... McNutt, R. L., Jr. (2012). Low-degree structure in Mercury's planetary magnetic field. *J. Geophys. Res.*, *117*(E12). doi: 10.1029/2012JE004159

Wessel, P., Smith, W. H. F., Scharroo, R., Luis, J. F., & Wobbe, F. (2013). Generic mapping tools: Improved version released. *Eos Trans. AGU*, *94*, 409–410. doi: 10.1002/2013EO450001

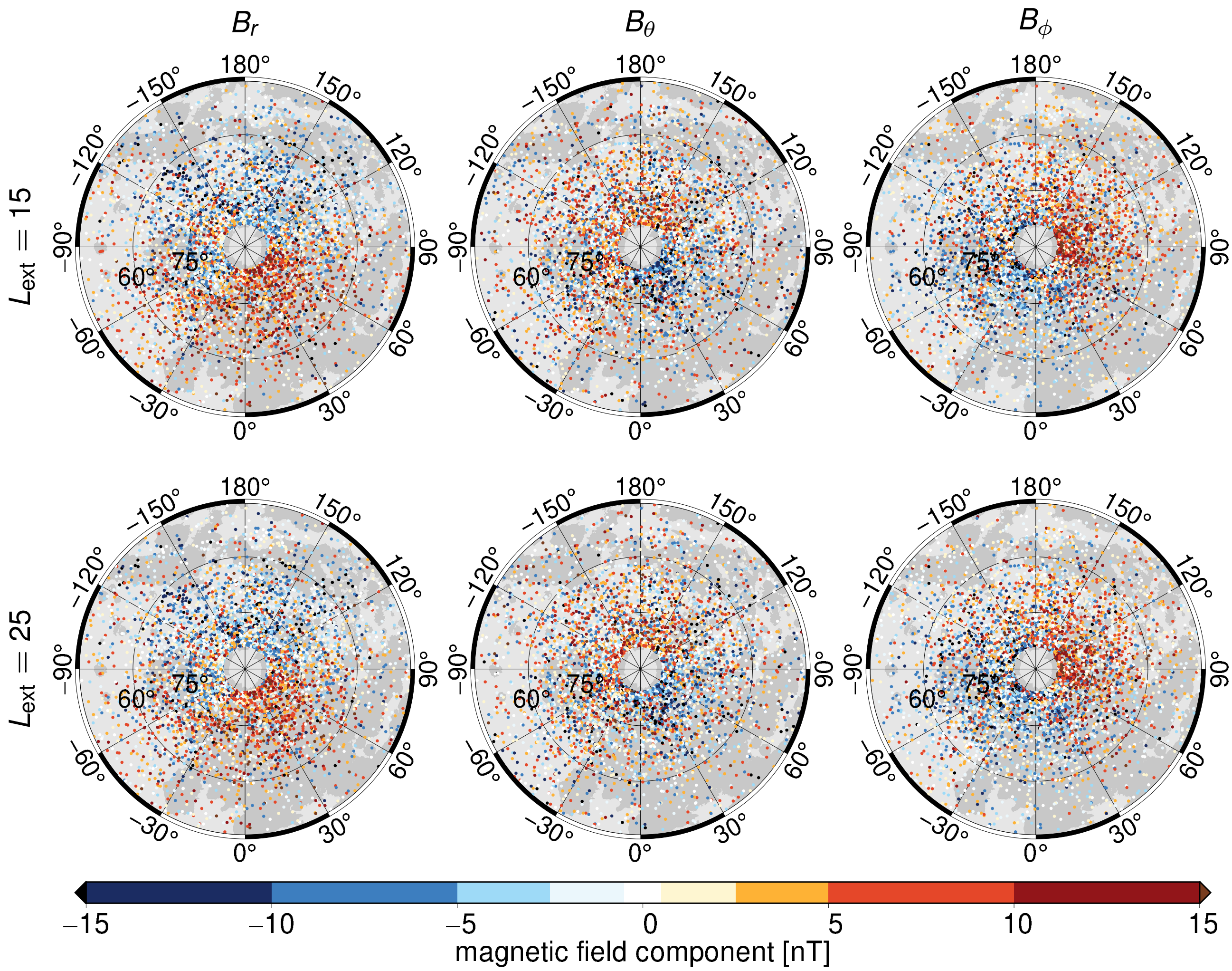

Figure S1. Filtering using different values for L_{ext}. Top row: $L_{ext} = 15$. Bottom row: $L_{ext} = 25$. Left column: radial component of the magnetic field. Center column: colatitudinal component. Right column: Longitudinal component.

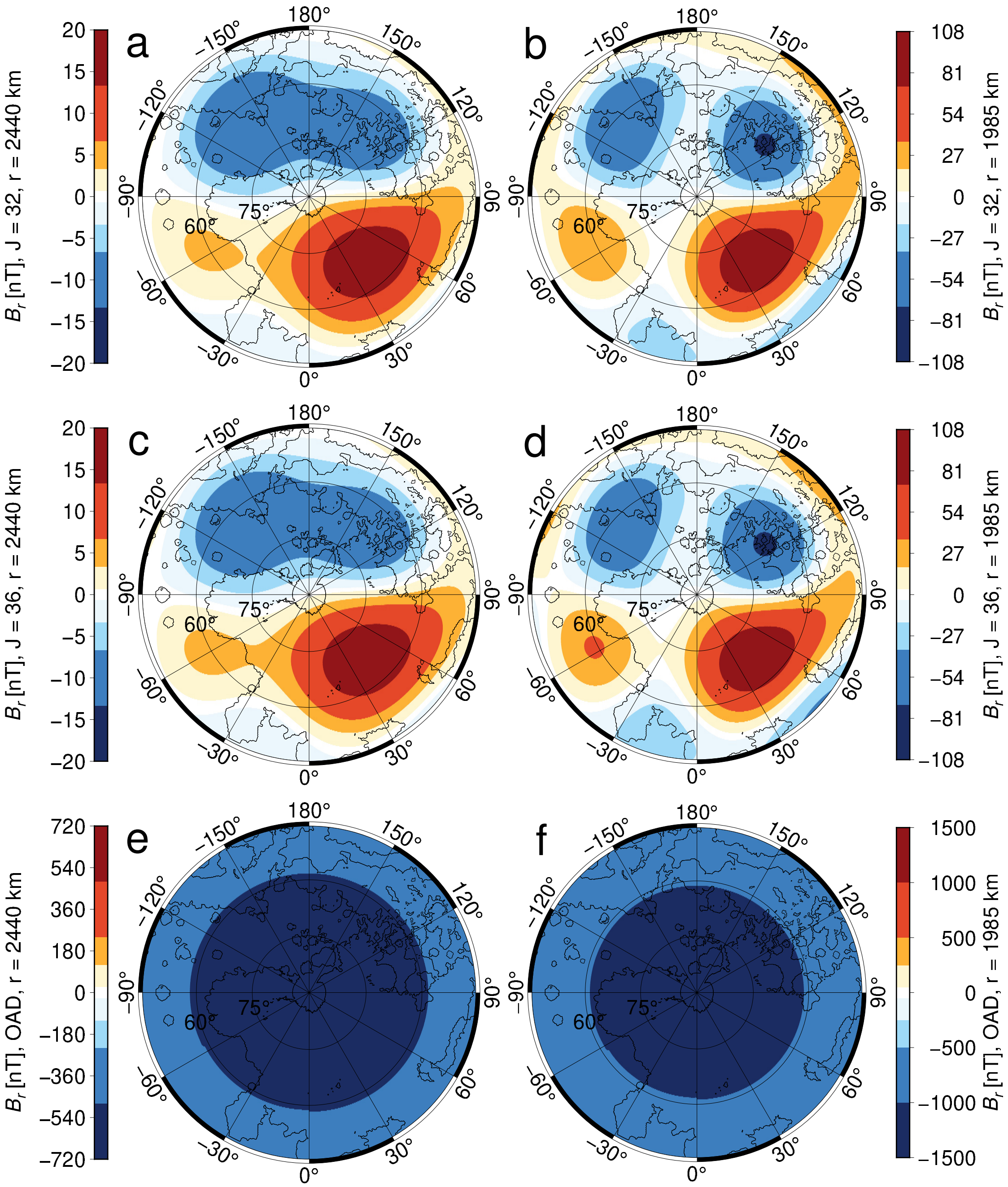

Figure S2. (a) Magnetic field model for $J = 32$ evaluated at Mercury's surface and (b) evaluated at the core-mantle boundary. (c) Magnetic field model for $J = 36$ evaluated at Mercury's surface and (d) evaluated at the core-mantle boundary. (e) Offset axial dipole (Anderson et al., 2012) at Mercury's surface and (f) at the core-mantle boundary.

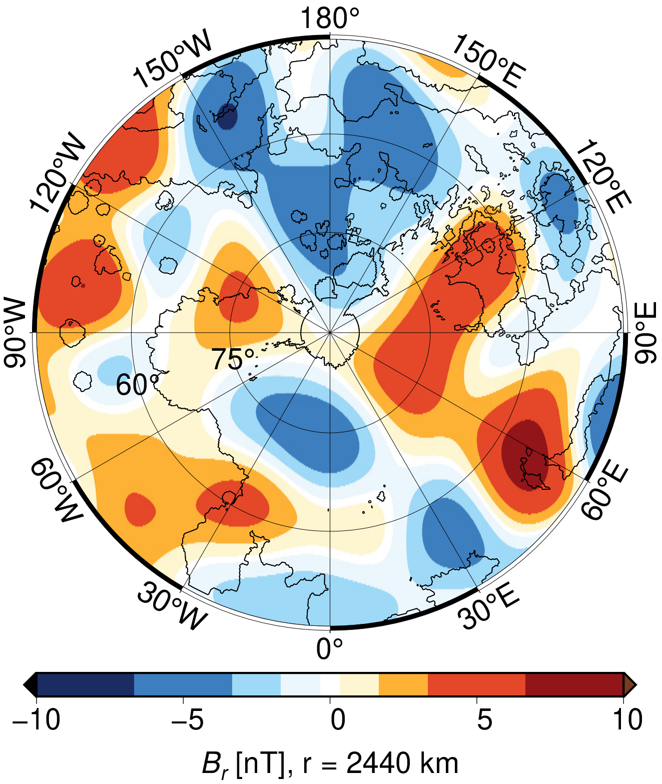

Figure S3. Crustal magnetic field model used for the synthetic experiment (Main article, Fig. 3d).

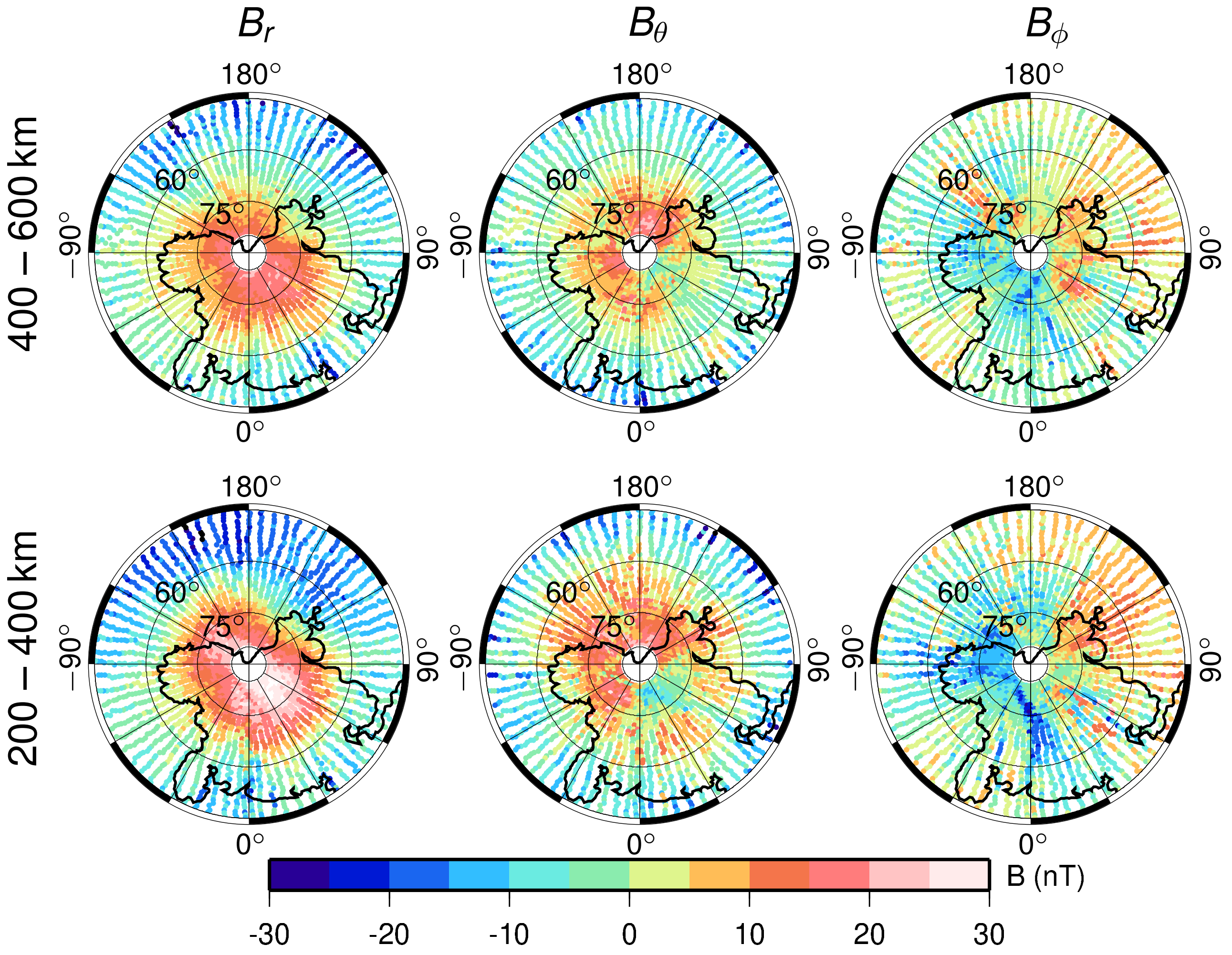

Figure S4. The radial (left), colatitudinal (center) and longitudinal (right) KT residuals for orbits with a disturbance index less than 50, in the MBF frame. Each panel shows the median residual computed from 10-second data in binned in 5° longitude by 1° latitude bins, and 200 km altitude bins for 200–400 km altitude (bottom row) and 400–600 km altitude (top row). Polar stereographic projections, extending from 45°N to the pole, grid lines every 15° latitude and 30° longitude. Solid black line shows the outline of the Northern Smooth Plains.

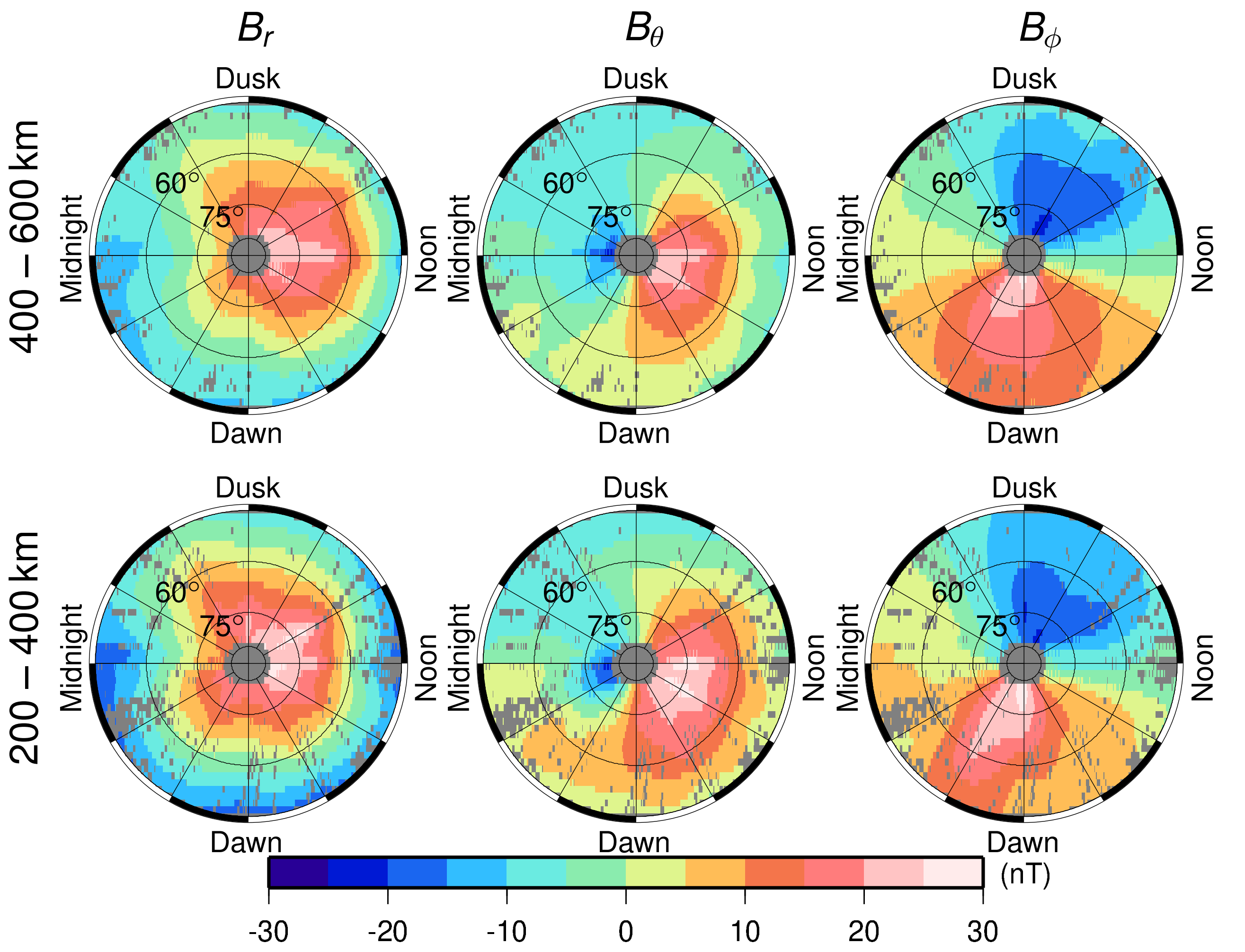

Figure S5. Smooth fits to the radial (left), colatitudinal (center) and longitudinal (right) KT residuals for the orbits in shown Fig. S4, in the aberrated, offset (Anderson et al., 2012) local time frame. The smooth fits were obtained for each field component and altitude bin separately, by binning into in 30° longitude by 5° latitude bins, and then using the blockmedian and surface commands in the Generic Mapping Tools (Wessel et al., 2013) package. Figure format as in Fig. S4, latitude is with respect to the magnetic equator.

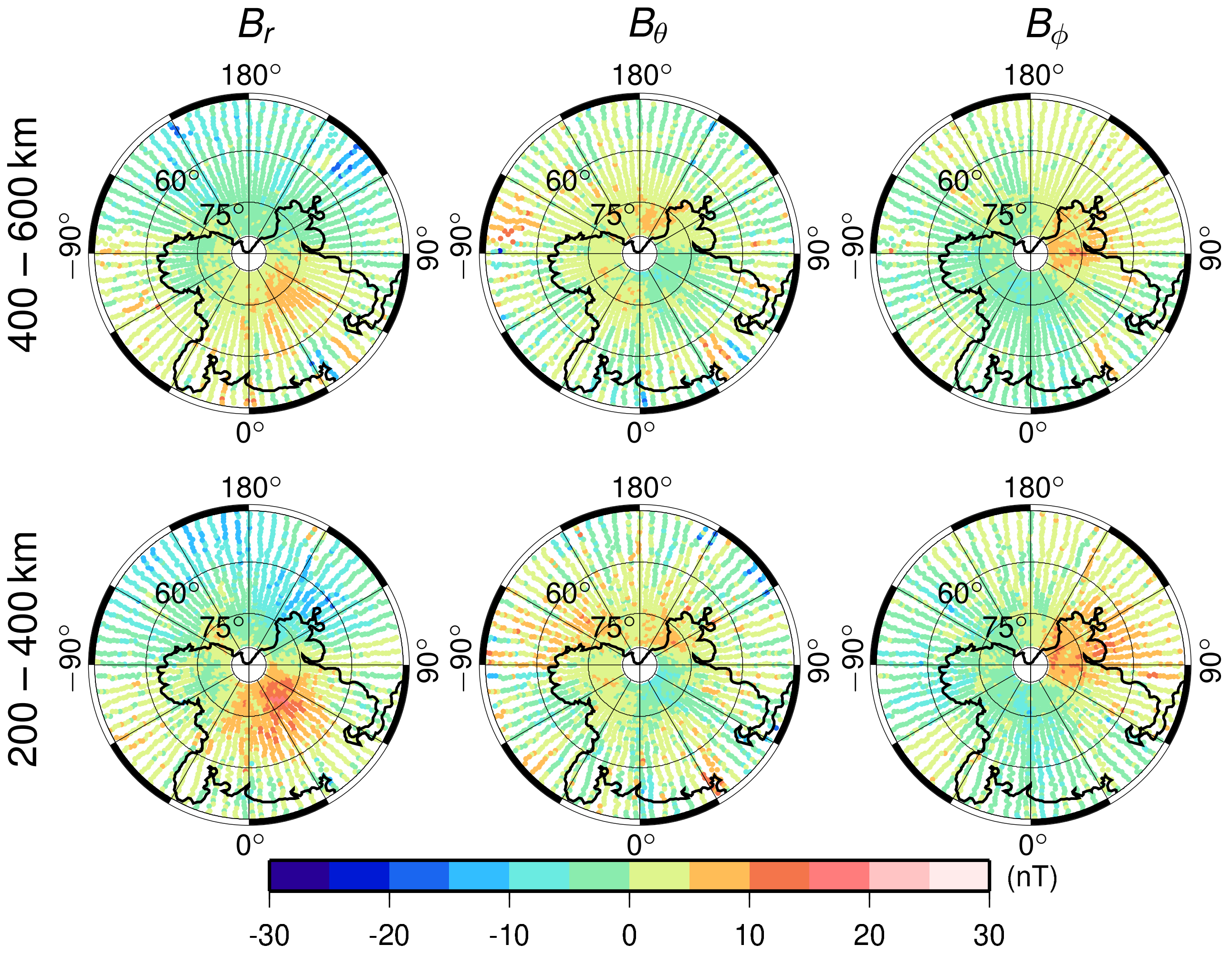

Figure S6. Filtered data in the MBF frame after subtraction of the smooth fits in Fig. S5. Color scale is the same as in Fig. S5 to show the clear reduction in amplitude after the filtering. Note the similar spatial patterns and amplitudes of the resulting signals as in Fig. 1 of the main text.
